# Experimental efficacy of vaccination of weaned piglets with a modified-live commercial PRRS virus vaccine against the challenge with a Spanish highly virulent PRRSV-1 strain

**DOI:** 10.1186/s40813-025-00423-y

**Published:** 2025-02-21

**Authors:** M. Cortey, M. Jiménez, L. Aguirre, J. M. Sánchez-Carvajal, J. Gómez-Laguna, I. Domingo-Carreño, H. Clilverd, M. Marcos, R. Menjon, S. Von Berg, E. Mateu

**Affiliations:** 1 Department of Animal Health and Anatomy, Universitat Autònoma de Barcelona, Travessera Dels Turons S/N, 08193 Cerdanyola del Vallès, Spain; 2 MSD Animal Health, Carbajosa de la Sagrada, Spain; 3 Department Anatomy and Comparative Pathology and Toxicology, Pathology and Immunology Group (UCO-PIG), UIC Zoonosis y Enfermedades Emergentes (ENZOEM), International Excellence Agrifood Campus ‘CeiA3”, University of Córdoba, 14014 Córdoba, Spain; 4 MSD Animal Health, Munich, Germany

**Keywords:** Porcine reproductive and respiratory syndrome virus, Virulence, Vaccine, Pig

## Abstract

**Background:**

In 2020, a highly virulent PRRSV-1 strain emerged in Spain and rapidly spread across the country. The purpose of the present study was to test in a piglet model whether a commercial PRRSV-1 modified live vaccine was able to confer protection against strain R1, a representative of the emerging clade. For that purpose, two groups of 26 piglets were either vaccinated intradermally or kept as controls; 42 days later, half of the animals in each group were intranasally challenged with the R1 strain. Then, animals were followed to assess the development of clinical signs (until 14 days post-challenge), lung lesions (10- and 35-days post-challenge), weight gains, viremia and nasal shedding and the immune response (anti PRRS virus nucleoprotein antibodies) by ELISA and virus specific-interferon-γ secreting cells by ELISPOT).

**Results:**

Challenge of naïve pigs resulted in high fever (up to 41.9 °C), lethargy and severely retarded growth (0.748 kg/day). In contrast, vaccinated/challenged pigs had less fever and for a shorter period, lower clinical scores and a higher average daily weight gain (0.940 kg/day), comparable to the unchallenged animals. At 10 days-post challenge, in naïve animals on average 49.1% of the lung was pneumonic (range 8–81%) while in vaccinated animals the average was 15.7% (4–41%). Duration of viremia was reduced in vaccinated animals and after 14 days post-challenge, most were negative by RT-qPCR. In contrast, 50% of the naïve/challenged pigs remained viremic at 35 days post-challenge. Vaccination induced rapid seroconversion and challenge of naïve animals resulted in 100% of ELISA-positive pigs by day 14 post-challenge. Regarding the development of IFN-γ responses, for vaccinated animals the frequencies increased until day 35 post-vaccination. After challenge, in vaccinated pigs, the peak of the R1-specific IFN-γ response was reached at 14 days and then the viremia ceased, although nasal shedding persisted in some vaccinated animals.

**Conclusions:**

In the present trial, vaccination resulted in improved clinical course, better weight gain and reduced viremia. At the peak of the infection, lung lesions were reduced in most animals although some individuals still had extensive pneumonia. In summary, vaccination was shown to provide partial but significant protection against the highly virulent R1 strain.

**Supplementary Information:**

The online version contains supplementary material available at 10.1186/s40813-025-00423-y.

## Background

Porcine reproductive and respiratory syndrome (PRRS) is one of the costliest diseases of pigs. PRRS is caused by two arteriviruses belonging to the Genus *Betaarterivirus*, PRRS virus 1 (PRRSV-1) and PRRSV-2. In general, PRRSV-2 strains have been considered to be more pneumovirulent than PRRSV-1 [15], although several reports indicated that PRRSV-1 may also contain highly virulent strains [3, 8, 22]. Recently, Martin-Valls et al. [13] reported the emergence of a highly virulent PRRSV-1 strain in Spain that has been commonly named Rosalia. The emerging strain in Spain most likely originated from a descendant of the PR40 clade reported by Canelli et al. [3] after undergoing several recombination events in Spain or elsewhere [13].

The introduction of the highly virulent PRRSV-1 in Spanish pig farms have resulted in a huge impact on sows and piglets [14]. On affected farms, abortion rates soar for months, often accompanied by a significant increase in mortality in pregnant sows. In addition, there is an increase in stillbirths, mummified sows, and pre-weaning mortality. Subsequently, in the nursery, mortality remains high (10–50%) for a long period of time, often for a year or even more [14].

Vaccination is one of the cornerstones in PRRS control. In sows it confers good clinical protection, resulting in a reduction in abortions, stillbirths, and mummies if the virus is introduced into the farm. In young pigs, vaccination helps reducing lung lesions, the duration of viremia and viral shedding. At a population level, it has been shown that for PRRSV-1, vaccination may help reducing the transmission of moderate virulent strains [16, 21]. However, the protection provided by vaccines is limited, as vaccinated animals can still be infected and transmit the infection. These limitations are particularly evident when the vaccine strain and the infecting strain are not genetically closely related [4, 6, 9]. Although heterologous cross-protection cannot be accurately predicted and many factors may influence it, in general, the more distant the vaccine and infecting strains are, the lower the degree of protection. With the emergence of new predominant PRRSV strains, particularly if highly virulent, the issue of cross-protection becomes crucial. The aim of the present study was to evaluate under experimental conditions the protection that vaccination with a commercial modified live vaccine can offer to pigs when they are challenged with the highly virulent PRRSV-1 Rosalia strain that recently emerged in Spain.

## Methods

**Fig. 1:**
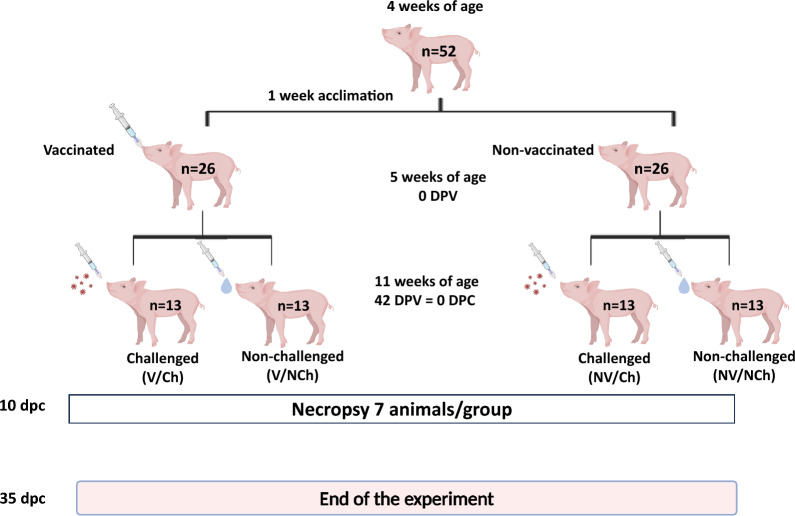
Design of the experimental infection, including the experimental groups: vaccinated and unchallenged (V/NCh), vaccinated and challenged (V/Ch), non-vaccinated and unchallenged (NV/NCh) and non-vaccinated and challenged (NV/Ch), age of the animals at every stage. Half of the animals were euthanized 10 DPC. The experiment ended at 35 DPC when the remaining animals were culled

Figure 1 depicts the design of the experimental infection, consisting in four groups of animals: vaccinated and non-challenged (V/NCh); vaccinated and challenged (V/Ch); non-vaccinated and non-challenged (NV/NCh) and, non-vaccinated and challenged (NV/Ch).

### Animals, housing and allocation.

Fifty-two 4-week-old (LandracexDuroc) piglets were purchased from a historically PRRSV-free farm were sows and piglets were seronegative.Animals were transported from the source farm to the animal facilities at the *Universitat Autònoma de Barcelona*. Pigs were inspected on arrival and were ear-tagged with numbered ear-tags (1–52). Animals were randomly divided in 4 groups of 13 animals using random numbers (random number function in Excel). Piglets were left to acclimatize for the next week in pens where temperature was controlled. Animals had ad libitum access to water and feed.

### Challenge virus

The isolate used in this study, designated as R1, was isolated from the serum of a piglet in a farm experiencing serious reproductive problems caused by PRRSV and increased mortality in the nurseries [14]. Production of the virus (passage n = 5) was performed in porcine alveolar macrophages (PAM). PAM were obtained by bronchoalveolar lavage of lungs of 4-week-old piglets from the same origin than the animals used in the experimental study. PAM used for growing the virus were tested for PRRSV, PCV2 and *Mycoplasma* by PCR and were found to be negative. Titration of the inoculum was performed in PAM and the titre was calculated as TCID_50_/ml using the Reed-Muench formula. The challenge strain R1 had been fully sequenced and is deposited in Genbank under accession number OM893828.

### Vaccination

After one week of acclimation (5 weeks of age), the treatment (vaccination/non-vaccination) was assigned by tossing a coin. Animals in vaccinated groups were administered with a 0.2 ml dose of a commercial modified live vaccine (MLV) Porcilis® PRRS via intradermal administration as recommended by the manufacturer using the IDAL® device of MSD Animal Health. Animals in the control unvaccinated groups received only 0.2 ml of the adjuvant of the vaccine. The vaccination day was considered day 0 of the experiment. The vaccine virus was titrated in MARC-145 cells for the various purposes it was used, with titres calculated as described above.

### Challenge

Challenge was performed on day 42 post-vaccination. Inoculation was carried out using an intranasal administration device (MAD Nasal ™, Teleflex). Each animal received a 2 ml dose (1 ml/nostril) of a cell culture supernatant of the strain R1, containing 10^5.4^ TCID_50_/ml.

### Clinical assessment

Clinical signs (respiratory signs, behavioural changes, other) and rectal temperatures were recorded from day -2 after challenge until day 14 post-challenge. The clinical score considered fever (0–4), respiratory signs (0–6), behavioural changes (0–4). Temperatures below 40 °C were considered within the normal range and afterwards each 0.5 °C increase accounted for 1 score point (40.00–40.50 °C, 1; 40.51–41.00, 2; etc.). For respiratory signs, 0 was normal respiratory function, 1 nasal discharge with no other signs, 2 sneezing, 3 coughing without evident dyspnea, 4 slight shortness of breath, 5 moderate shortness of breath and 6 severe shortness of breath, abdominal breathing and open mouth. For the behavioural changes, 0 was normal behaviour, 1 was apathetic but responsive to stimulation, 2 prostration and only respond when touched, 3 prostrated unresponsive but conscious and 4, coma. Data were registered and scored in the appropriate clinical record datasheets.

### Weighing

Animals were weighed on the day of vaccination, on the challenge day and then weekly until the end of the experiment at day 35 post-challenge using a calibrated scale. Data were registered in appropriate datasheets.

### Pathology

At least six animals per group were sacrificed at 10 days post-challenge (DPC) using an overdose of anaesthetics to assess the development of lung lesions. Immediately after the euthanasia and the exsanguination of the animals, lungs were removed from the thoracic cavity and the presence of pneumonia and other lung lesions was determined. The macroscopic pneumonic score (proportion of affected lung) was calculated over 100 using a standard scoring system [7] that considered the proportion of pneumonic tissue in each lobe (ventrally and dorsally). Then, samples of the cranial and caudal parts of cranial left lung lobe and from the caudal left lung lobe were taken and submerged in 10% buffered formalin for the histopathological analysis. Histological lung sections were blindly examined by two experienced pathologists and an estimated score of the severity of the interstitial pneumonia as well as suppurative bronchopneumonia was given as previously described by Halbur et al. [7] and Rodríguez-Gómez et al. [20], respectively. Furthermore, lung lesion scoring system included an additional point to the presence of proliferative necrotizing pneumonia (PNP). The final score comprised the sum of both, the interstitial pneumonia score and the bronchopneumonia score, as well as PNP being 9 points the maximum possible score. The presence of additional lesions such as perivascular infiltrate, peribronchial or peribronchiolar infiltrate, haemorrhages or re-epithelialisation of alveoli was also registered but not included in the lung lesion score. At the end of the study (35 DPC), the remaining animals were euthanized, and the assessment of lung lesions was performed as above. Lung samples were taken for histopathological analyses as well. Histological preparations were examined blindly by two experienced pathologists and the averaged scores were used.

### Blood sampling

Animals were sampled on days 0, 7, 14, 21, 28, 42 (0 DPC), 46 (4 DPC), 49 (7 DPC), 52 (10 DPC), 56 (14 DPC), 63 (21 DPC), 70 (28 DPC) and 77 (35 DPC). On all the mentioned days blood samples were taken by jugular venopuncture using siliconized tubes (BD Vacutainer®). On days, 0, 14, 28, 42, 49, 56, 63, 70 and 77 blood samples from 24 randomly selected animals (6 per group) were also collected using heparinized tubes. Nasal swabs were taken from the same 24 animals on days 42, 46, 49, 52, 56, 63, 70 and 77.

### Haematological analyses

Heparinized blood samples collected weekly after challenge and analysed with a XN 1000 1A analyzer. The haematological parameters analysed included total red and white blood cell counts, haematocrit, haemoglobin-related indices, monocyte, lymphocyte, neutrophil, eosinophil, and basophil counts along with their relative proportions, as well as platelet counts.

#### Virological analyses

The detection of PRRSV in blood or nasal swabs (Virocult, VWR Spain) was performed using the LSI VETMAX PRRSEUNA 2.0 kit (Thermofisher). RNA was extracted using the MagMAx Core kit (Thermofisher) using a KingFisher Flex extraction robot (Thermofisher) following the supplier recommendations.

#### Serological analyses.

Sera were analysed first for the presence of anti-PRRSV antibodies by means of the PRRS X3 Ab kit (Idexx Laboratories) according to the manufacturer’s protocol.

## ELISPOT

PBMC were separated form heparinized blood by gradient centrifugation using Histopaque® 1.077 (Merck). Cells (250,000/well) were seeded onto 96-well PDVF plates (Merck) coated with anti pig-Interferon gamma antibody P2G10 (BD Bisociences) and stimulated with either PRRSV (Porcilis® or R1 challenge strain) at 0.1 m.o.i. or mock-stimulated with RPMI culture medium. PHA-stimulated wells were used as positive controls (10 µg/ml). Development of the spots was assessed by adding the biotinylated anti-porcine IFN-γ antibody P2C11 (BD Biosciences) and AEC (Mabtech) for revealing the reaction. Spots were visually counted and examined under magnification. For the counting of the virus-specific IFN-γ secreting cells (IFN-SC), the counts in the mock-stimulated cultures were subtracted from the counts of virus-stimulated cells. Frequencies of IFN-SC were expressed as number of cells per million PBMC.

### Statistical analyses

Statistical analyses were performed using Graphpad Prism 10.4 (available at www.GraphPad.com). Comparison of means between groups was performed by using the non-parametric Kruskal–Wallis test, comparisons between proportions were calculated using the *χ*^2^ test with Fisher’s exact test. Relative risk was used to compare the proportion of animals with high fever in vaccinated and control challenge groups. Area under the curve for viremia and viral shedding was calculated considering the average 40-Ct values for each timepoint (including both positive and negative samples) using the area under the curve calculation utility in Graphpad.

## Results

### Clinical follow-up

**Fig. 2:**
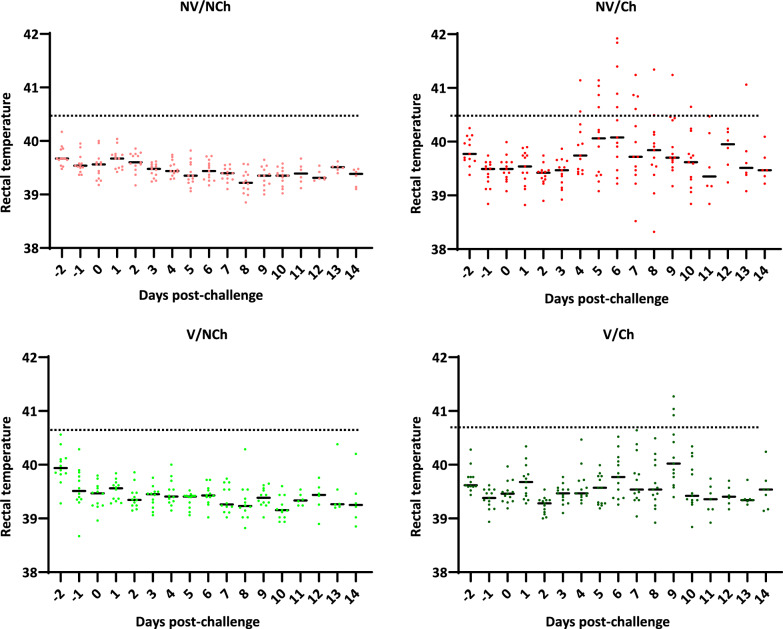
Individual records of the rectal temperature in the four groups of study from day -2 to 14 DPC. The bars depict the median for each day and group. V = vaccinated; NV = non-vaccinated; Ch = challenged; NCh = non challenged

One animal (V/NCh) had to be removed from the trial because of an unresolved umbilical hernia. Apart from this, animals did not show any disease sign before the challenge. After challenge, clinical signs were observed in all infected groups but with different severity. Regarding rectal temperatures (Fig. 2), NV/Ch showed the highest temperatures during infection with a record high of 41.92 °C (6 days post-challenge, DPC). It is worth noting a high variability in the individual response. Significant differences for the rectal temperatures (*p* < 0.05) between groups were observed from 4 to 10 DPC (Supplementary material 1). For the vaccinated and challenged group (V/Ch), rectal temperatures were significantly lower (*p* < 0.05) than those of the NV/Ch group at 5- and 6-DPC, and the average temperatures were similar between V/Ch and NV/Ch from 7 DPC onwards. Notably, in the V/Ch animals the development of fever started later and less animals were affected with a shorter duration of the feverish period. Thus, in the NV/Ch group the highest proportion of animals with fever (temperature > 40.5 °C) was 38.5% at 6 DPC while at that same day only 7.7% of the V/Ch showed fever (Supplementary material 2).

Regarding other clinical signs, the most common observation was lethargy. Besides, some animals showed laboured breathing. NV/NCh animals did not show clinical sign during the observation period. In contrast, for the NV/Ch group, clinical scores started to increase by day 4 DPC reaching the highest values by 7 DPC. In this group all animals scored above zero at least for one day (Supplementary material 3). Vaccinated animals had less clinical signs and for less time. When the results were compared day by day (Supplementary material 4) the NV/Ch had significantly higher scores than the controls from 4 to 8 DPC. Regarding the V/Ch animals, they had less clinical signs and were not different from the unchallenged controls although on 6–8 DPC they were also like the NV/Ch group.

### Weight gains

Animals were weighed when vaccinated, the day of the challenge and every week afterwards. Average weekly weight gains were calculated, showing that after challenge, NV/Ch animals suffered a severe retardation in the weight gain rates compared to unchallenged controls (Table 1). Thus, for the 0–35 DPC period, the NV/Ch animals gained on average about a 25% less weight than the NV/NCh controls. This difference was higher for the first 14 days post-challenge. While the NV/NCh gained on average 1.003 kg/day in those two weeks, the NV/Ch animals gained only 0.656 kg/day (34.6% reduction). In contrast, the average daily weight gain of the V/Ch in the same 14 day-period post-challenge was 0.978 kg/day (a 49% increase compared to the NV/Ch controls and just 2.5% less than the controls). Most of differences in growth rates accumulated in the first 14 days after challenge (Supplementary material 5).

**Table 1:** Total and average daily weight gains for the whole period of challenge

Group	Total weight gain 0–35 DPC (Kg)	Average daily weight gains 0–35 DPC (Kg)
NV/NCh	34.2 ± 8.1	0.977^a^
NV/Ch	26.2 ± 6.1	0.748^b^
V/NCh	35.0 ± 6.7	1.000^a^
V/Ch	32.9 ± 12.8	0.940^a,b^

Superscripts with different letters indicate statistically significant (p < 0.05) differences

### Lung lesions

**Fig. 3:**
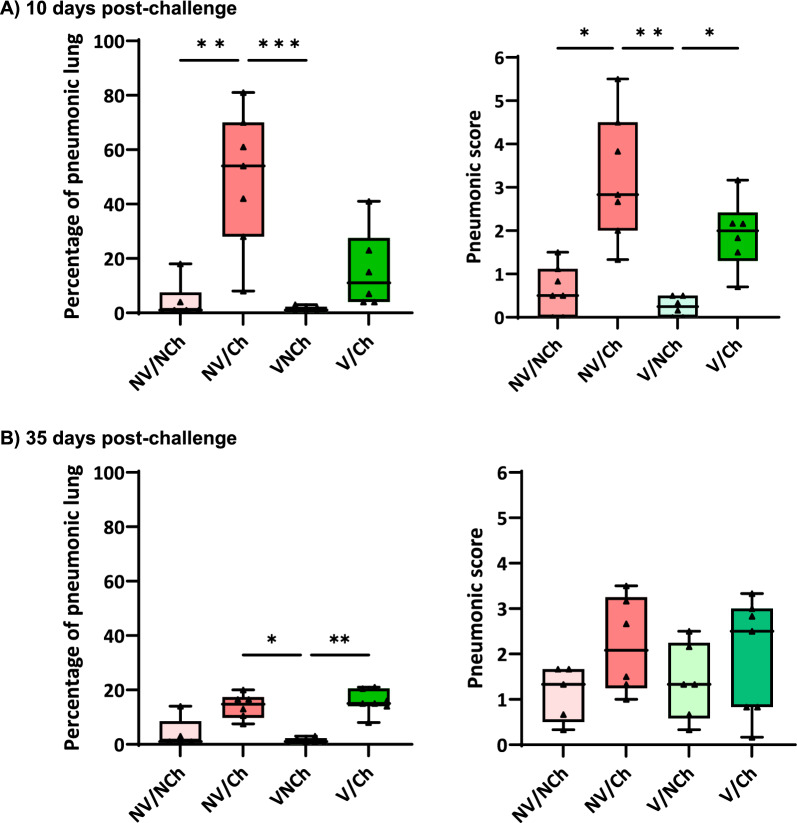
Macroscopic (left) and microscopic (right) scores at 10 days-post challenge (A) and 35 days post-challenge. V = vaccinated; NV = non-vaccinated; Ch = challenged; NCh = non challenged. *p < 0.05; **p < 0.01; ***p < 0.001; ****p < 0.0001

**Fig. 4:**
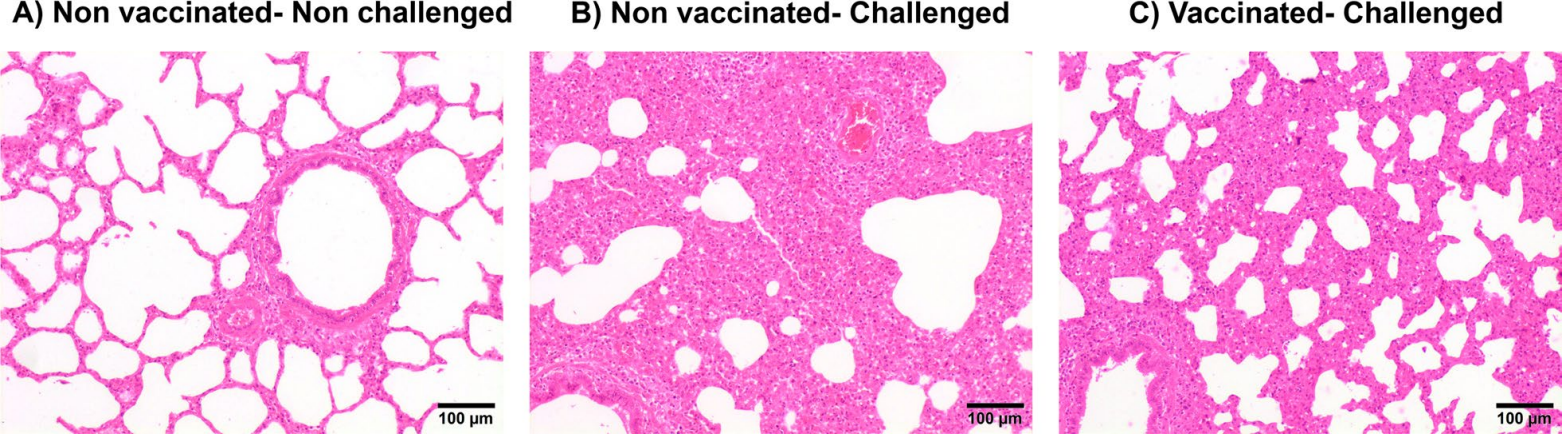
Examples of the lung lesions in the different groups. Photomicrographs illustrate the severity of interstitial pneumonia in each group at 10 DPC. **A** Non-Vaccinated-Non challenged pig; **B** Non vaccinated- Challenged and **C** Vaccinated—Challenged. Vaccinated-non-challenged is not shown since it was similar to the non- vaccinated- non-challenged ones. Hematoxylin and eosin. Bar, 100 μm

**Fig. 5:**
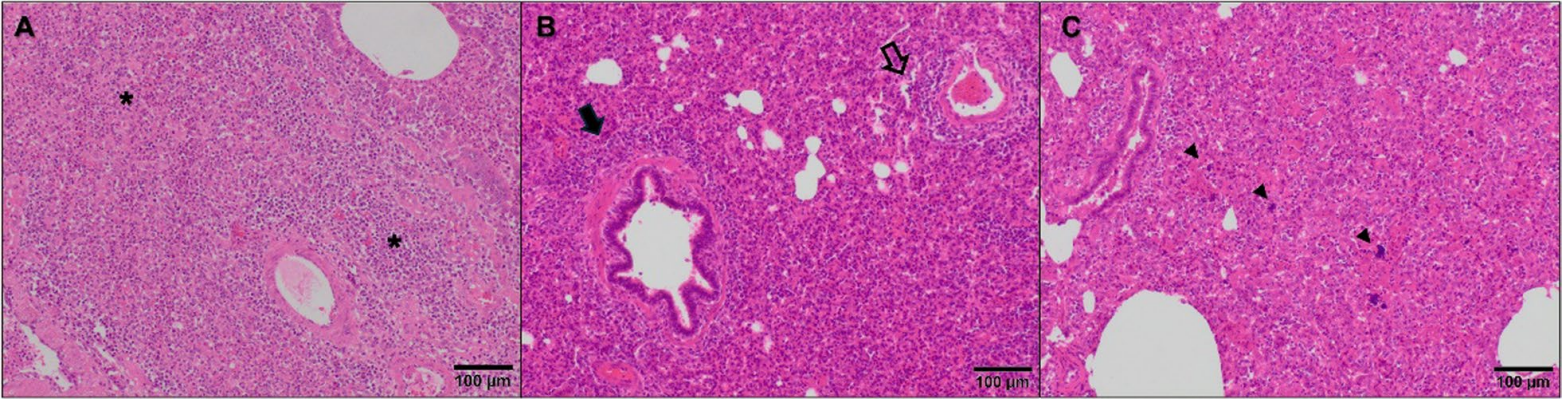
Characteristic microscopic lung lesions in challenged piglets. Photomicrographs of the medial lung lobe of a NV/Ch piglet illustrate suppurative bronchopneumonia (**A**, asterisk), inflammatory infiltrate by lymphocytes, macrophages and monocytes surrounding the bronchi and bronchioles (**B**, arrow), and a proliferative necrotizing pneumonia in a representative challenged piglet (**C**, arrowhead) at 10 DPC. Hematoxylin and eosin. Bar, 100 μm

Lung lesions were examined at 10 DPC and at the end of the experiment. Necropsies performed at 10 DPC revealed the development of serious pneumonic lesions in the non-vaccinated animals, both at macroscopic and microscopic level (Figs. 3 and 4). Animals in the NV/Ch group scored on average 49.1% (range 8–81%) of the lung area affected by pneumonia compared to 15.7% (4–41%) in vaccinated animals while the proportion of pneumonic lung was scored between 1.2% and 4.2% in the non-challenged groups. Regarding the microscopic lesion scores, the NV/Ch group had the highest average values (p < 0.05) compared to the non-challenged groups. In the histopathological evaluation, severe interstitial pneumonia characterized by thickening of the alveolar septa due to infiltration by lymphocytes, macrophages, and occasional presence of plasma cells, was observed in the animals inoculated with the highly virulent Rosalia strain. Additionally, suppurative bronchopneumonia was observed in several lung sections from Rosalia-infected piglets at 10 DPC (Fig. 5A). One of the main characteristic lesions was a marked inflammatory infiltration surrounding the bronchi and bronchioles (Fig. 5B), as well as multifocal proliferative necrotizing pneumonia (Fig. 5C). This severe form of interstitial pneumonia was exclusively observed in NV/Ch animals at 10 DPC.

At 35 DPC the proportion of pneumonic lung surface was still higher in the challenged groups (14.0% and 15.64% in the NV/Ch and V/Ch group, respectively) compared to the non-challenged groups (1.17% and 4%). Microscopic lung lesion scores showed no differences between groups with high individual variability.

### Haematological analyses.

Animals were examined every week after challenge to determine significant variations in the main haematological parameters. Of these, the number of lymphocytes was severely affected in the challenged groups. At 0 DPC all animals have similar counts of lymphocytes per microliter (µl) of blood (ranging between 9.8 and 12.5 × 10^3^ lymphocytes/µl) and showed a considerable variation within each group. At 7 DPC both inoculated groups (NV/Ch and V/Ch) suffered a significant (*p* < 0.05) decrease in the proportion of circulating lymphocytes (6.83 ± 2.12 × 10^3^ lymphocytes/µl and 6.43 ± 1.96 × 10^3^ lymphocytes/µl for NV/Ch and V/Ch, respectively) compared to the non-challenged ones (9.84 ± 1.27 × 10^3^ lymphocytes/µl and 10.89 ± 1.57 × 10^3^ lymphocytes/µl for NV/NCh and V/Ch, respectively). These differences reverted to normal at 14 DPC and no differences were observed afterwards. Supplementary Fig. 6 shows the evolution of lymphocytes counts over time. No significant changes in monocytes, neutrophils or any other haematological parameter were observed.

### Viremia and nasal shedding.

**Fig. 6:**
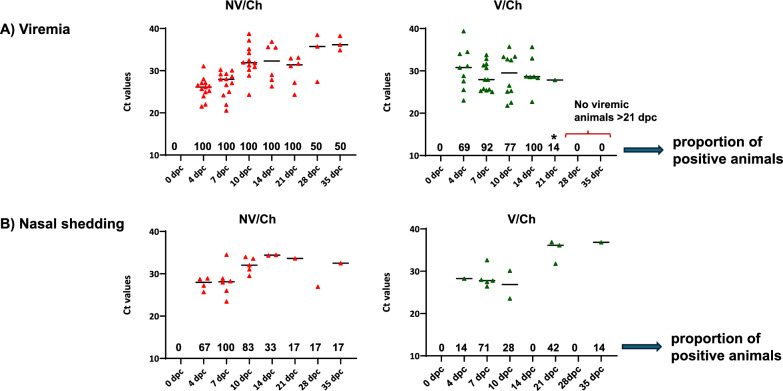
Viremia and nasal shedding in challenged groups. The graphs show the individual Ct values and the proportion of RT-qPCR PRRSV-positive animals at each timepoint for viremia (**A**) and nasal shedding (**B**). The number over the date shown in the X-axis indicates the proportion of positive animals. V = vaccinated; NV = non-vaccinated; Ch = challenged; dpc = days post-challenge. Statistical significance for the proportion of positive animals in V/Ch versus NV/Ch is indicated with an asterisk (*p* < 0.05)

The examination of sera by RT-qPCR showed that in the NV/Ch group viremia persisted up to 35 DPC in half of the examined animals (Fig. 6). In contrast, for the V/Ch group none of the examined animals was viremic after 21 DPC, resulting in a shortening of viremia in vaccinated pigs of up to 14 days compared to non-vaccinated ones. The calculation of the AUC for the viremia also showed a clear reduction when comparing unvaccinated and vaccinated groups (256.2 *vs.* 110.4 in NV/Ch and V/Ch, respectively).

Regarding nasal shedding, virus was found in nasal secretions up to 35 DPC in both vaccinated and unvaccinated groups although the AUC was significantly lower in V/Ch compared to the non-vaccinated pigs (43.7 vs 124.8, respectively).

### Serological analyses

**Fig. 7:**
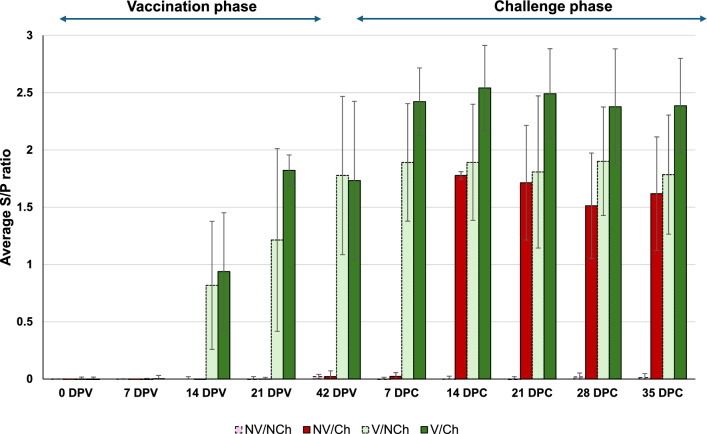
Serological evolution as determined by ELISA. The graph shows the average S/P ratio values for each group as determined using the Idexx ELISA during the vaccination and challenge phases. V = vaccinated; NV = non-vaccinated; Ch = challenged; NCh = non-challenged. DPC = days post-challenge

As expected, animals were all PRRSV seronegative at the beginning of the study. Vaccination induced seroconversion in most animals between 7- and 21-days post administration of the vaccine. In the NV/Ch group, inoculation with the highly virulent PRRSV-1 induced rapid seroconversion and by 14 DPC all animals were seropositive. In the V/Ch animals, inoculation with the wild-type virus resulted in a significant increase in the S/P ratios by 7 DPC (1.73 ± 0.27 before inoculation vs. 2.42 ± 0.29 at 7 DPC, *p* < 0.01). Figure 7 summarize the results.

## ELISPOT

**Fig. 8:**
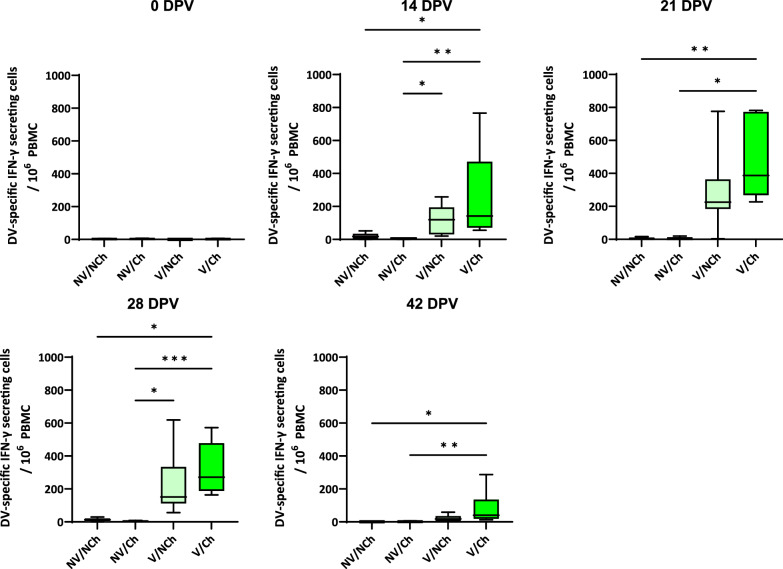
IFN-γ ELISPOT using the vaccine virus (strain DV) as antigen during the vaccination phase. The graph shows the frequencies of vaccine-specific IFN-γ secreting cells per million PBMC during the vaccination phase. V = vaccinated; NV = non-vaccinated; Ch = challenged; NCh = non-challenged. DPV = days post-vaccination

**Fig. 9:**
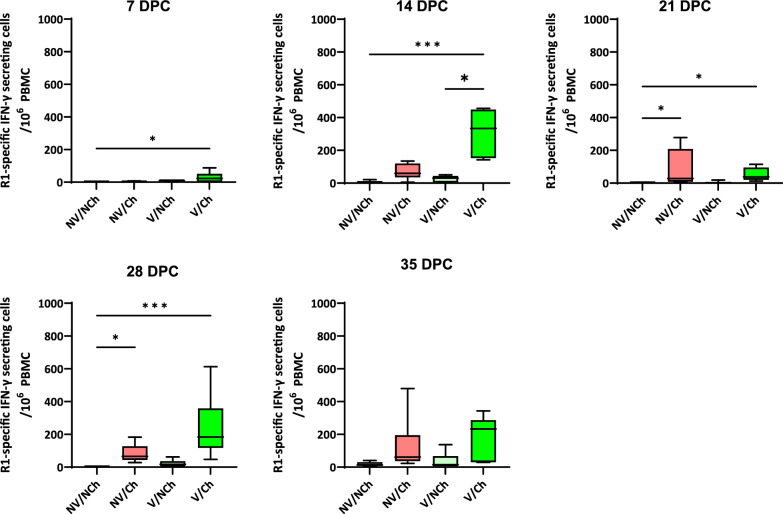
IFN-γ ELISPOT using the highly virulent strain R1 as antigen during the challenge phase. The graph shows the frequencies of R1-specific IFN-γ secreting cells per million PBMC during the challenge phase. V = vaccinated; NV = non-vaccinated; Ch = challenged; NCh = non-challenged. DPV = days post-vaccination

The results of the ELISPOT analyses against the vaccine (DV, accession number KJ127878) and the challenge (R1, accession number OM893828) strains (Figs. 8 and 9, respectively) showed that after vaccination, vaccinated animals developed a strong cell-mediated response against the vaccine virus, reaching more than 770 IFN-SC per 10^6^ peripheral blood mononuclear cells (PBMC) in the highest responder (21 DPV). Non vaccinated animals produced responses that were like those in the unstimulated cultures. Afterwards, the cell-mediated response contracted until the moment of the challenge. Challenge produced a significant booster of the response in the vaccinated group against the vaccine strain but also against the highly virulent strain when it was used as stimulus (14 DPC), indicating the existence of cross-reactive epitopes.

During the challenge phase the specific response of IFN-SC against the highly virulent PRRSV-1 R1 strain was also examined. It is worth noting that for the V/Ch group, the average frequencies of IFN-γ secreting cells –(IFN-SC) obtained in response to challenge R1 or the vaccine DV strain at the peak response during the challenge phase (311 ± 136 vs. 350 ± 163 at 14 DPC, n.s.) were similar. In contrast, the non-vaccinated animals developed relatively low frequencies, well below those of the vaccinated animals. Interestingly, after 21 DPC frequencies of IFN-SC against the vaccine DV strain were very low. Results obtained in the ELISPOT with the DV vaccine strain after challenge are shown in supplementary material 7.

## Discussion

In the present study, the efficacy of a commercial MLV to protect against the development of the disease caused by a highly virulent PRRSV-1 isolate that emerged in Spain was evaluated. This new highly virulent PRRSV-1 clade (commonly called Rosalia) was originally detected in Spain in 2020 [13] and since then, has spread all over the country. The analysis of its genome indicates that its origin can be traced back to the descendants of the PR40 strain described in Italy by Canelli et al. [3]. However, the original Rosalia R1 isolate harbour several genome segments resulting from recombination events, some of them probably with local Spanish strains. The impact of these highly virulent isolates on the productivity of the infected farms is huge [14], and although precise statistics are lacking, it is considered partially responsible for the decrease in the supply of pigs to the Spanish slaughterhouses in these last years. According to the Spanish Ministry of Agriculture and Fisheries, the number of slaughtered pigs in Spain decreased by more than 5 million between 2021 and 2023, namely a 9.1% reduction despite that the census was only reduced by 0.19% in the same period (data available at: https://www.mapa.gob.es/es/ganaderia/temas/produccion-y-mercados-ganaderos/sectores-ganaderos/porcino/indicadoreseconomicos.aspx).

Vaccination is one of the tools that can be used to alleviate the impact of the infection. In previous papers, it was shown that the tested vaccine was able to confer partial but significant protection against viremia and clinical signs against other virulent PRRSV-1 strains such as PR40 or Lena [4, 23]. In general terms, a growing number of experiments supports the notion that commercial MLV are able to confer a certain level of protection against highly virulent PRRSV strains, resulting in a better outcome in vaccinated individuals compared to non-vaccinated ones [2, 11, 12], although protection is only partial.

In the present trial, the NV/Ch animals developed high fever, evident clinical signs and suffered a significant decrease in the weight gain rates. In contrast, V/Ch had less days with fever or clinical signs. As in previous studies [4] the impact of the infection upon weight gains was clear with a reduction of 0.229 kg/day of gain weight in the NV/Ch group compared to the NV/NCh animals and 0.252 kg/day compared to the V/NCh (*p* < 0.05). The weight gains of the V/Ch animals were not significantly different than those of the NV/NCh although still suffer some reduction. This is a very remarkable feature as weight gain is a critical parameter to consider for pig productivity.

To note, the challenged groups showed a significant drop in blood lymphocyte counts on day 7 post-challenge that was reversed by day 14. A similar fact was found by Canelli et al. [4] studying the PR40 strain, although in their case, lymphopenia in NV/Ch animals did not reverse until 21 DPC. The reasons for these discrepancies remain unclear but may be attributed to differences in the vaccination route (intradermal in our study versus intramuscular in theirs), the age at challenge (11 weeks versus 9 weeks), or the strain used. The observed reduction in blood lymphocyte counts could stem from diverse mechanisms, including precursor depletion or increased cellular trafficking to infection sites. Moreover, in contrast to Canelli’s findings, red blood cell counts, hematocrit, and hemoglobin levels in our study remained unaffected.

Regarding viremia and nasal shedding, our results agree with the partial protection reported by Canelli et al. [4] and Trus et al. [23], who tested the same vaccine against other highly virulent PRRSV-1 strains,the Italian PR40 strain from subtype 1, and the Belarusian strain Lena from subtype 3, respectively. In the case of the present study, in most animals viremia ceased after day 14 DPC although nasal shedding persisted in some animals until 35 DPC. This reduction in the viremia took place after the peak of the Rosalia-specific IFN-γ response at 14 DPC, suggesting the existence of common epitopes in the Rosalia and DV strains that were relevant to protection. Unfortunately, the incapability to adapt Rosalia strain to grow on MARC-145 made impossible to test the presence of cross-reacting neutralizing antibodies. However, based on Canelli’s results using the PR40 strain [4], DV was expected to produce very low titres of cross-reacting neutralizing antibodies against Rosalia. The prolonged nasal shedding observed would agree with the notion that that virulent PRRSV strains may show enhanced replication in the nasal mucosa [5]. It can also suggest that the induction tissue resident memory T cells was less intense than the induction of effector or central memory T cells. It is worth noting that for the first 21 days after challenge the ELISPOT results obtained using the strain DV or the challenge strain R1 were similar suggesting that during the first weeks post-challenge there was an expansion of the memory T cells induced by vaccination. At 28 and 35 DPC, the ELISPOT frequencies for the DV strain were very low while frequencies of IFN-SC for the R1 strain were much higher suggesting that the specific R1 response of IFN-SC required 3–4 weeks to develop.

Protection against the development of lung lesions showed a great individual variation although in general the lowest scores among challenged animals corresponded to the vaccinated ones. Thus, at 10 DPC, the difference on the average proportions of pneumonic lung between NV/Ch and V/Ch was 33% although the scores ranged from 8 to 81% in NV/Ch to 4 to 41% in V/Ch. This same variation was seen when recording the clinical signs. The reasons for this huge variability are not fully clear but may rely on the genetic individual background. Several reports have shown that breed and several SNP can be related to the resilience to the infection [1, 10, 18, 24]. In our case, the used animals were Landrace x Duroc crossbred pigs coming from different litters and were not matched for specific genetic traits.

In the present study, the clinical manifestations observed in NV/Ch animals, including fever and other clinical signs, were comparable to those reported in previous studies involving virulent PRRSV-1 strains such as Lena or PR40 [4, 8]. Notably, in our case, viremia persisted in half of the animals for up to 35 days, compared to 28 DPC in studies with Lena or PR40. In the present study, nasal shedding was also detected up to 35 DPC in some animals, which exceeds the duration reported for Lena or PR40. These findings suggest a similar level of clinical severity, albeit with somewhat prolonged viral shedding, which could potentially impact transmissibility. Regarding lung lesions, the severity observed in this study aligns with the findings of Rawal et al. [17] for several 1-4-4 L1C isolates, except for L5C, which was associated with more severe lesions. In terms of vaccine efficacy, our results are consistent with those reported by Renson et al. [19] for strain Lena and Canelli et al. [4] for strain PR40, demonstrating a reduction in fever, an improvement in weight gain, and a moderate reduction in viremia.

Finally, some aspects of the experimental design and the interpretation of the results should be evaluated with care. The first one relates to the time allowed between vaccination and challenge, namely 42 days. Commonly, most vaccination/challenge for PRRSV are performed with a challenge at 28 or, at maximum, 35 days post-vaccination. In our case, the trial was designed as a proof-of-concept to examine the potential of the vaccine for protecting against the Rosalia strain based on the development of memory cells and minimizing the interference by effector cells developed during the expansion of the immune response after priming and, at the same time, to minimize the proportion of animals positive for the vaccine virus at the moment of the challenge. As shown by the results, at 42 DPV, the IFN-γ response was already contracting and there were no viremic animals, fulfilling thus the initial aim. However, a challenge at an earlier time post-vaccination could have resulted in more or less protection than in the present case. The impossibility to adapt the strain used for challenge to continuous cell lines avoided the testing of neutralizing antibodies. However, as mentioned above, it is unlikely that the vaccine could have induced significant amounts of neutralizing antibodies against the challenge strain.

## Conclusions

In summary, in the context of the present study the assayed MLV induced significant, clinical, pathological and virological protection against the challenge with a Hv-PRRSV-1 Spanish strain. This finding supports the notion that MLV vaccination can be beneficial against this very virulent strain if time enough for the development of the immune response is allowed.

## Supplementary Information

Supplementary material 1. Comparison of daily temperature records per group. The figure depicts the daily individual records of rectal temperatures for the whole period of observation (-2 to 14 DPC) with statistical comparison between groups. V = vaccinated, NV= non-vaccinated; Ch = challenged, NCh= non challenged. *p<0.05; **p<0.01; ***p<0.001; ****p<0.0001

Supplementary material 2. Proportion of animals with rectal temperatures >40.5°C per day. V = vaccinated, NV= non-vaccinated; Ch = challenged

Supplementary material 3. Distribution of the individual daily clinical scores per group. V = vaccinated, NV= non-vaccinated; Ch = challenged, NCh= non challenged

Supplementary material 4. Comparison of daily clinical scores per group. The figure depicts the daily individual clinical scores for the 4-10 DPC period with statistical comparison between groups. V = vaccinated, NV= non-vaccinated; Ch = challenged, NCh= non challenged. *p<0.05; **p<0.01; ***p<0.001; ****p<0.0001

Supplementary material 5. Daily weight gains per group. The figure depicts the daily gain weights per groups with statistical comparison. V = vaccinated; NV= non-vaccinated; Ch = challenged; NCh= non challenged. *p<0.05; **p<0.01; ***p<0.001; ****p<0.0001

Supplementary material 6. Evolution of lymphocyte counts in blood per group after challenge. The figure depicts weekly lymphocyte counts per group after challenge with statistical comparison between groups. Differences were only observed at 7 days post-challenge (DPC). V = vaccinated, NV= non-vaccinated; Ch = challenged, NCh= non challenged. *p<0.05

Supplementary material 7. Evolution of the frequencies of IFN-γ secreting cells after challenge observed in ELISPOT using the vaccine strain as stimulus. V = vaccinated, NV= non-vaccinated; Ch = challenged, NCh= non challenged. *p<0.05; **p<0.01; ***p<0.001; ****p<0.0001

## Data Availability

The datasets analysed during the current study are available from the corresponding author on reasonable request.

## Abbreviations

AEC: 3-Amino-9-ethylcarbazole
Ch: Challenged
Ct: Cycle threshold
DPC: Days post-challenge
DPV: Days post-vaccination
ELISA: Enzyme-linked immunosorbent assay
ELISPOT: Enzyme-linked immunosorbent spot assay
IFN-γ: Interferon gamma
IFN-SC: Interferon gamma secreting cells
MLV: Modified live vaccine
NCh: Non challenged
NV: Non vaccinated
PAM: Porcine alveolar macrophages
PBMC: Peripheral blood mononuclear cells
PCV2: Porcine circovirus 2
PHA: Phytohaemagglutinin
PRRS: Porcine reproductive and respiratory syndrome
PRRSV: Porcine reproductive and respiratory syndrome virus
RPMI: Roswell Park memorial Institute medium
RT-qPCR: Reverse transcription quantitative polymerase chain reaction
V: Vaccinated

## References

[1] Boddicker N, Waide EH, Rowland RR, Lunney JK, Garrick DJ, Reecy JM, Dekkers JC. Evidence for a major QTL associated with host response to porcine reproductive and respiratory syndrome virus challenge. J Anim Sci. 2012;90(6):1733–46. 10.2527/jas.2011-4464. 22205662 10.2527/jas.2011-4464

[2] Bonckaert C, van der Meulen K, Rodríguez-Ballarà I, Pedrazuela Sanz R, Martinez MF, Nauwynck HJ. Modified-live PRRSV subtype 1 vaccine UNISTRAIN® PRRS provides a partial clinical and virological protection upon challenge with East European subtype 3 PRRSV strain Lena. Porcine Health Manag. 2016;2:12. 10.1186/s40813-016-0029-y. 28405438 10.1186/s40813-016-0029-y PMC5382438

[3] Canelli E, Catella A, Borghetti P, Ferrari L, Ogno G, De Angelis E, Corradi A, Passeri B, Bertani V, Sandri G, Bonilauri P, Leung FC, Guazzetti S, Martelli P. Phenotypic characterization of a highly pathogenic Italian porcine reproductive and respiratory syndrome virus (PRRSV) type 1 subtype 1 isolate in experimentally infected pigs. Vet Microbiol. 2017;210:124–33. 10.1016/j.vetmic.2017.09.002. 29103681 10.1016/j.vetmic.2017.09.002

[4] Canelli E, Catella A, Borghetti P, Ferrari L, Ogno G, De Angelis E, Bonilauri P, Guazzetti S, Nardini R, Martelli P. Efficacy of a modified-live virus vaccine in pigs experimentally infected with a highly pathogenic porcine reproductive and respiratory syndrome virus type 1 (HP-PRRSV-1). Vet Microbiol. 2018;226:89–96. 10.1016/j.vetmic.2018.10.001. 30389048 10.1016/j.vetmic.2018.10.001

[5] Frydas IS, Nauwynck HJ. Replication characteristics of eight virulent and two attenuated genotype 1 and 2 porcine reproductive and respiratory syndrome virus (PRRSV) strains in nasal mucosa explants. Vet Microbiol. 2016;182:156–62. 10.1016/j.vetmic.2015.11.016. 26711043 10.1016/j.vetmic.2015.11.016

[6] Huang Y, Li Z, Li J, Yibo-Kong YL, Mah CK, Liu G, Yu B, Wang K. Efficacy evaluation of three modified-live PRRS vaccines against a local strain of highly pathogenic porcine reproductive and respiratory syndrome virus. Vet Microbiol. 2019;229:117–23. 10.1016/j.vetmic.2018.12.016. 30642586 10.1016/j.vetmic.2018.12.016

[7] Halbur PG, Paul PS, Frey ML, et al. Comparison of the pathogenicity of two US porcine reproductive and respiratory syndrome virus isolates with that of the Lelystad virus. Vet Pathol. 1995;32(6):648–60. 10.1177/030098589503200606. 8592800 10.1177/030098589503200606

[8] Karniychuk UU, Geldhof M, Vanhee M, Van Doorsselaere J, Saveleva TA, Nauwynck HJ. Pathogenesis and antigenic characterization of a new East European subtype 3 porcine reproductive and respiratory syndrome virus isolate. BMC Vet Res. 2010;6:30. 10.1186/1746-6148-6-30. 20525333 10.1186/1746-6148-6-30 PMC2898778

[9] Labarque G, Reeth KV, Nauwynck H, Drexler C, Van Gucht S, Pensaert M. Impact of genetic diversity of European-type porcine reproductive and respiratory syndrome virus strains on vaccine efficacy. Vaccine. 2004;22(31–32):4183–90. 10.1016/j.vaccine.2004.05.008. 15474708 10.1016/j.vaccine.2004.05.008

[10] Lough G, Hess A, Hess M, Rashidi H, Matika O, Lunney JK, Rowland RRR, Kyriazakis I, Mulder HA, Dekkers JCM, Doeschl-Wilson A. Harnessing longitudinal information to identify genetic variation in tolerance of pigs to Porcine reproductive and respiratory syndrome virus infection. Genet Sel Evol. 2018;50(1):50. 10.1186/s12711-018-0420-z. 30355341 10.1186/s12711-018-0420-z PMC6201485

[11] Madapong A, Saeng-Chuto K, Chaikhumwang P, Tantituvanont A, Saardrak K, Pedrazuela Sanz R, Miranda Alvarez J, Nilubol D. Immune response and protective efficacy of intramuscular and intradermal vaccination with porcine reproductive and respiratory syndrome virus 1 (PRRSV-1) modified live vaccine against highly pathogenic PRRSV-2 (HP-PRRSV-2) challenge, either alone or in combination with of PRRSV-1. Vet Microbiol. 2020;244:108655. 10.1016/j.vetmic.2020.108655. 32402335 10.1016/j.vetmic.2020.108655

[12] Madapong A, Saeng-Chuto K, Boonsoongnern A, Tantituvanont A, Nilubol D. Cell-mediated immune response and protective efficacy of porcine reproductive and respiratory syndrome virus modified-live vaccines against co-challenge with PRRSV-1 and PRRSV-2. Sci Rep. 2020;10(1):1649. 10.1038/s41598-020-58626-y. 32015495 10.1038/s41598-020-58626-y PMC6997162

[13] Martín-Valls GE, Cortey M, Allepuz A, Illas F, Tello M, Mateu E. Description of a new clade within subtype 1 of *Betaarterivirus suid 1* causing severe outbreaks in Spain. Microbiol Resour Announc. 2022;11(7):e0030422. 10.1128/mra.00304-22. 35652666 10.1128/mra.00304-22 PMC9302161

[14] Martín-Valls GE, Cortey M, Allepuz A, Illas F, Tello M, Mateu E. Introduction of a PRRSV-1 strain of increased virulence in a pig production structure in Spain: virus evolution and impact on production. Porcine Health Manag. 2023;9(1):1. 10.1186/s40813-022-00298-3. 36597152 10.1186/s40813-022-00298-3 PMC9811746

[15] Martínez-Lobo FJ, Díez-Fuertes F, Segalés J, García-Artiga C, Simarro I, Castro JM, Prieto C. Comparative pathogenicity of type 1 and type 2 isolates of porcine reproductive and respiratory syndrome virus (PRRSV) in a young pig infection model. Vet Microbiol. 2011;54(1–2):58–68. 10.1016/j.vetmic.2011.06.025. 10.1016/j.vetmic.2011.06.025 21831539

[16] Pileri E, Gibert E, Soldevila F, García-Saenz A, Pujols J, Diaz I, Darwich L, Casal J, Martín M, Mateu E. Vaccination with a genotype 1 modified live vaccine against porcine reproductive and respiratory syndrome virus significantly reduces viremia, viral shedding and transmission of the virus in a quasi-natural experimental model. Vet Microbiol. 2015;175(1):7–16. 10.1016/j.vetmic.2014.11.007. 25439650 10.1016/j.vetmic.2014.11.007

[17] Rawal G, Almeida MN, Gauger PC, Zimmerman JJ, Ye F, Rademacher CJ, Armenta Leyva B, Munguia-Ramirez B, Tarasiuk G, Schumacher LL, Aljets EK, Thomas JT, Zhu JH, Trexel JB, Zhang J. In vivo and in vitro characterization of the recently emergent PRRSV 1-4-4 L1C variant (L1C5) in comparison with other PRRSV-2 lineage 1 isolates. Viruses. 2023;15(11):2233. 10.3390/v15112233. 38005910 10.3390/v15112233 PMC10674456

[18] Reiner G, Willems H, Pesch S, Ohlinger VF. Variation in resistance to the porcine reproductive and respiratory syndrome virus (PRRSV) in Pietrain and Miniature pigs. J Anim Breed Genet. 2010;127(2):100–6. 10.1111/j.1439-0388.2009.00818.x. 20433517 10.1111/j.1439-0388.2009.00818.x

[19] Renson P, Fablet C, Le Dimna M, Mahé S, Touzain F, Blanchard Y, Paboeuf F, Rose N, Bourry O. Preparation for emergence of an Eastern European porcine reproductive and respiratory syndrome virus (PRRSV) strain in Western Europe: Immunization with modified live virus vaccines or a field strain confers partial protection. Vet Microbiol. 2017;204:133–40. 10.1016/j.vetmic.2017.04.021. 28532792 10.1016/j.vetmic.2017.04.021

[20] Rodríguez-Gómez IM, Sánchez-Carvajal JM, Pallarés FJ, Mateu E, Carrasco L, Gómez-Laguna J. Virulent Lena strain induced an earlier and stronger downregulation of CD163 in bronchoalveolar lavage cells. Vet Microbiol. 2019;235:101–9. 10.1016/j.vetmic.2019.06.011. 31282367 10.1016/j.vetmic.2019.06.011

[21] Rose N, Renson P, Andraud M, Paboeuf F, Le Potier MF, Bourry O. Porcine reproductive and respiratory syndrome virus (PRRSv) modified-live vaccine reduces virus transmission in experimental conditions. Vaccine. 2015;33(21):2493–9. 10.1016/j.vaccine.2015.03.040. 25820061 10.1016/j.vaccine.2015.03.040

[22] Sinn LJ, Klingler E, Lamp B, Brunthaler R, Weissenböck H, Rümenapf T, Ladinig A. Emergence of a virulent porcine reproductive and respiratory syndrome virus (PRRSV) 1 strain in Lower Austria. Porcine Health Manag. 2016;2:28. 10.1186/s40813-016-0044-z. 28405454 10.1186/s40813-016-0044-z PMC5382404

[23] Trus I, Bonckaert C, van der Meulen K, Nauwynck HJ. Efficacy of an attenuated European subtype 1 porcine reproductive and respiratory syndrome virus (PRRSV) vaccine in pigs upon challenge with the East European subtype 3 PRRSV strain Lena. Vaccine. 2014;32(25):2995–3003. 10.1016/j.vaccine.2014.03.077. 24709589 10.1016/j.vaccine.2014.03.077

[24] You X, Li G, Lei Y, Xu Z, Zhang P, Yang Y. Role of genetic factors in different swine breeds exhibiting varying levels of resistance/susceptibility to PRRSV. Virus Res. 2023;326:199057. 10.1016/j.virusres.2023.199057. 36731630 10.1016/j.virusres.2023.199057 PMC10194364

