# Supplementary figures and images for: Experimental efficacy of vaccination of weaned piglets with a modified-live commercial PRRS virus vaccine against the challenge with a Spanish highly virulent PRRSV-1 strain
Source: Porcine Health Manag. 2025 Feb 21;11:10. doi: 10.1186/s40813-025-00423-y (PMC11846179; doi:10.1186/s40813-025-00423-y)

## Slide 1
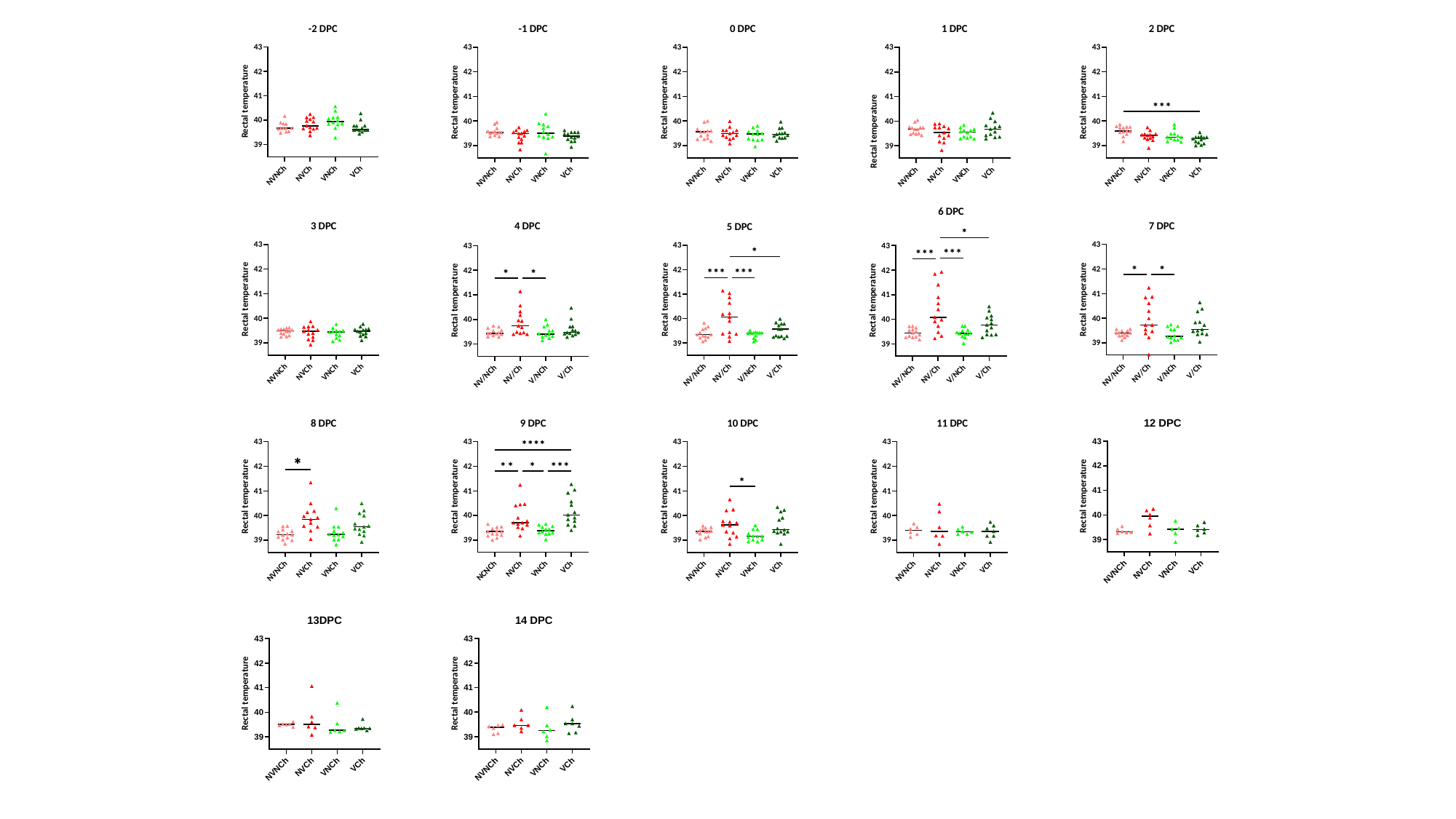

Supplement: Supplementary file 1 — Supplementary material 1. Comparison of daily temperature records per group. The figure depicts the daily individual records of rectal temperatures for the whole period of observation (-2 to 14 DPC) with statistical comparison between groups. V = vaccinated, NV= non-vaccinated; Ch = challenged, NCh= non challenged. *p<0.05; **p<0.01; ***p<0.001; ****p<0.0001 [file 40813_2025_423_MOESM1_ESM.pptx]

## Slide 1
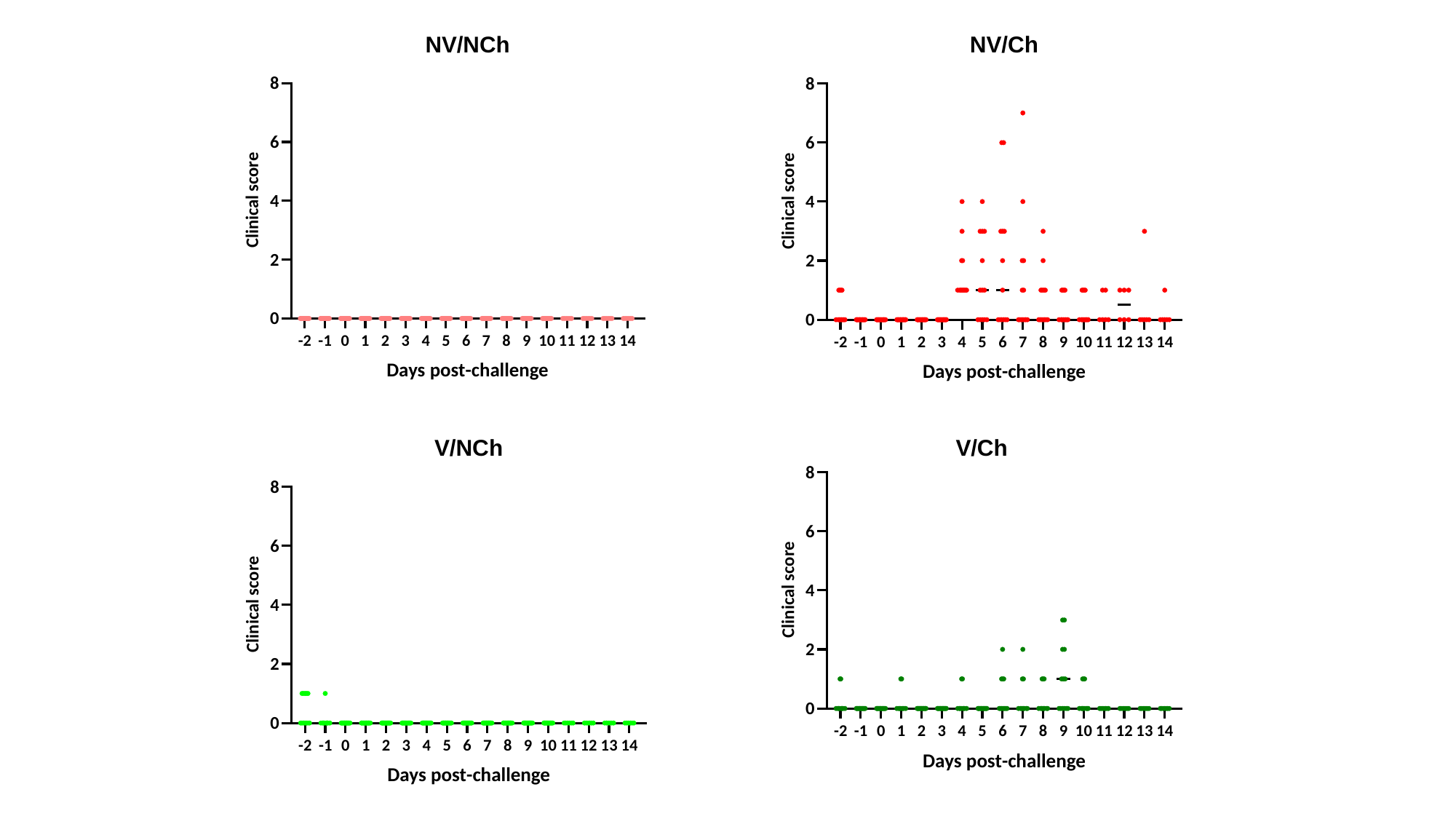

Supplement: Supplementary file 3 — Supplementary material 3. Distribution of the individual daily clinical scores per group. V = vaccinated, NV= non-vaccinated; Ch = challenged, NCh= non challenged [file 40813_2025_423_MOESM3_ESM.pptx]

## Slide 1
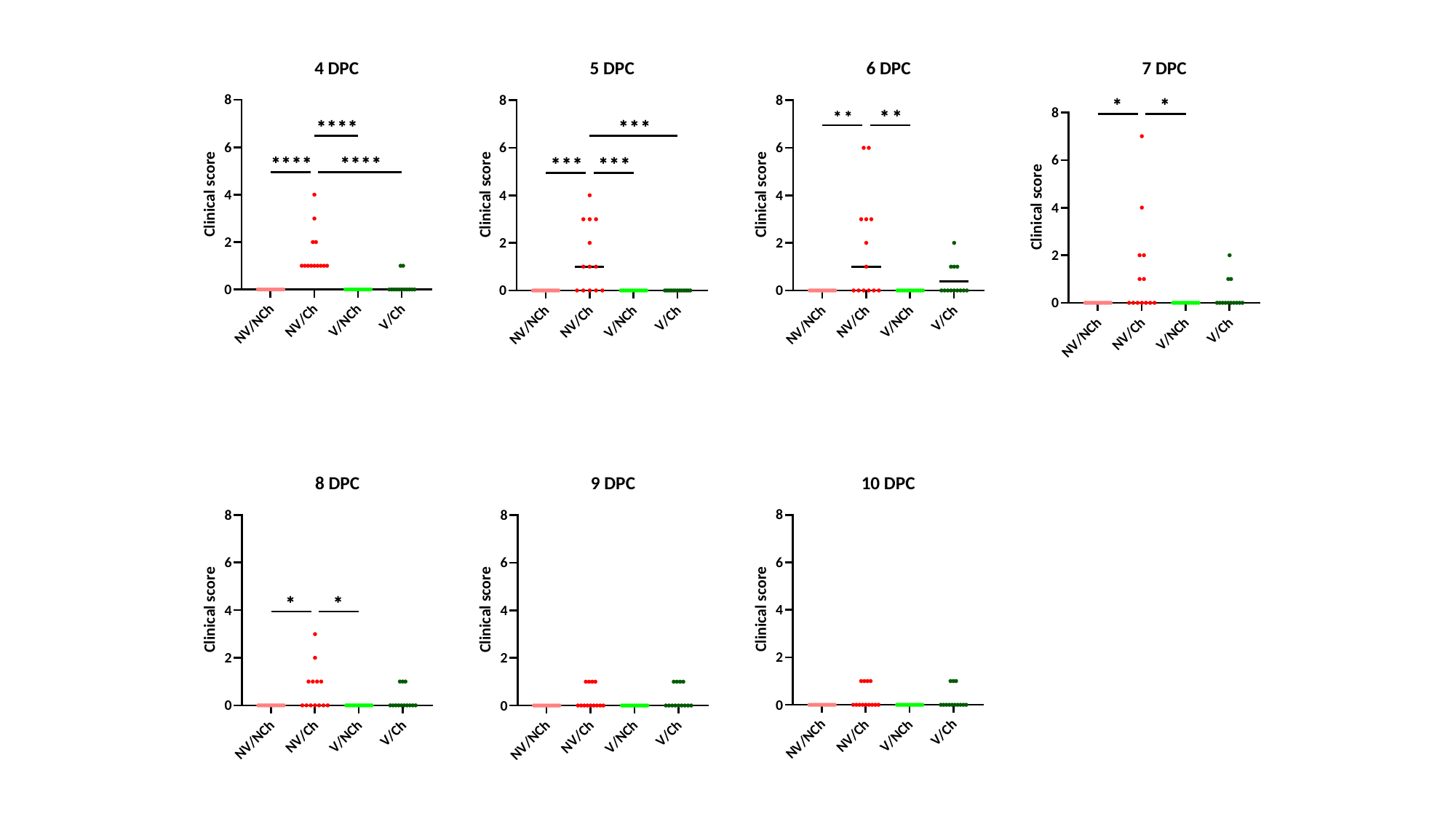

Supplement: Supplementary file 4 — Supplementary material 4. Comparison of daily clinical scores per group. The figure depicts the daily individual clinical scores for the 4-10 DPC period with statistical comparison between groups. V = vaccinated, NV= non-vaccinated; Ch = challenged, NCh= non challenged. *p<0.05; **p<0.01; ***p<0.001; ****p<0.0001 [file 40813_2025_423_MOESM4_ESM.pptx]

## Slide 1
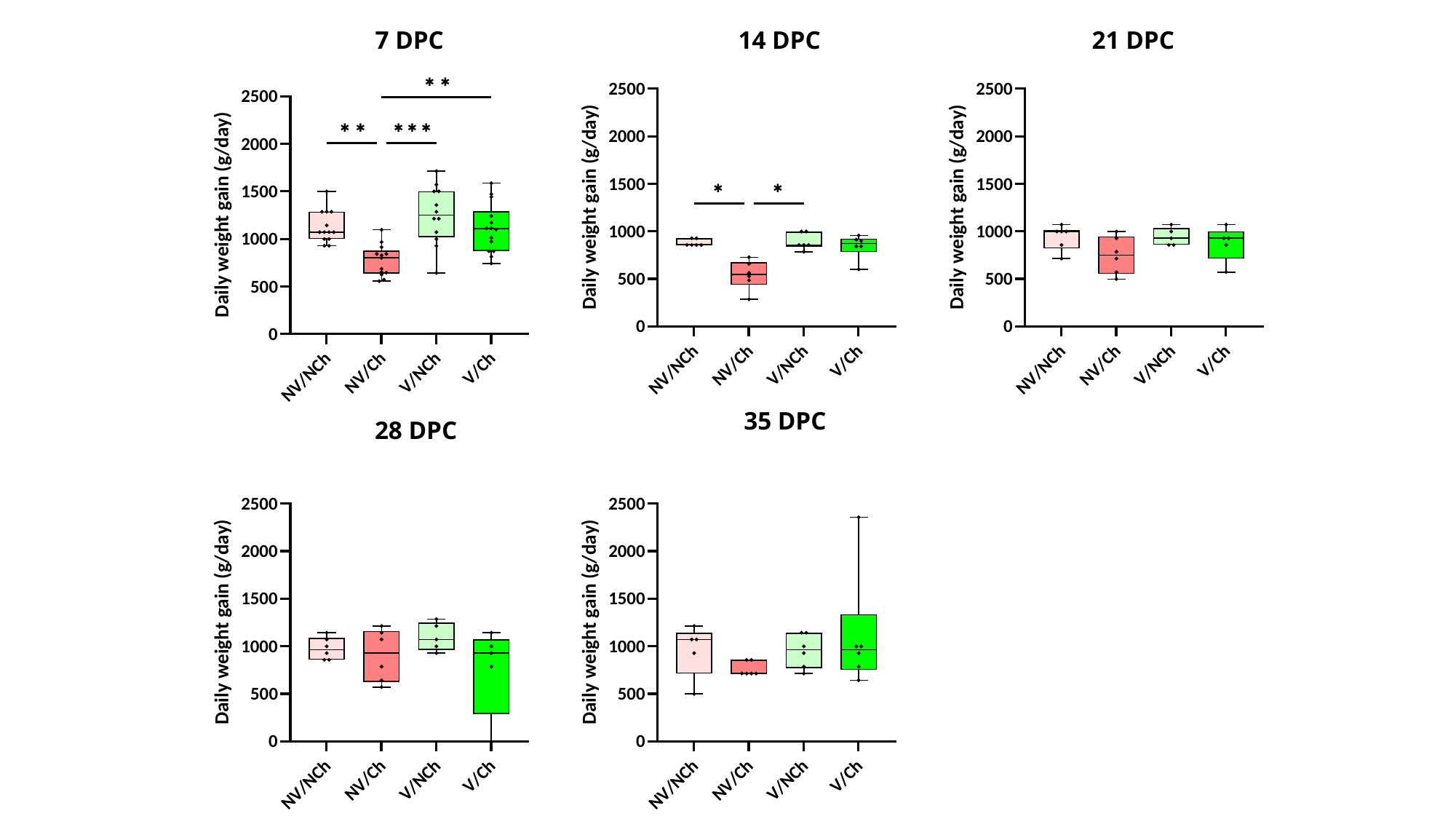

7 DPC
14 DPC
21 DPC
35 DPC
28 DPC

Supplement: Supplementary file 5 — Supplementary material 5. Daily weight gains per group. The figure depicts the daily gain weights per groups with statistical comparison. V = vaccinated; NV= non-vaccinated; Ch = challenged; NCh= non challenged. *p<0.05; **p<0.01; ***p<0.001; ****p<0.0001 [file 40813_2025_423_MOESM5_ESM.pptx]

## Slide 1
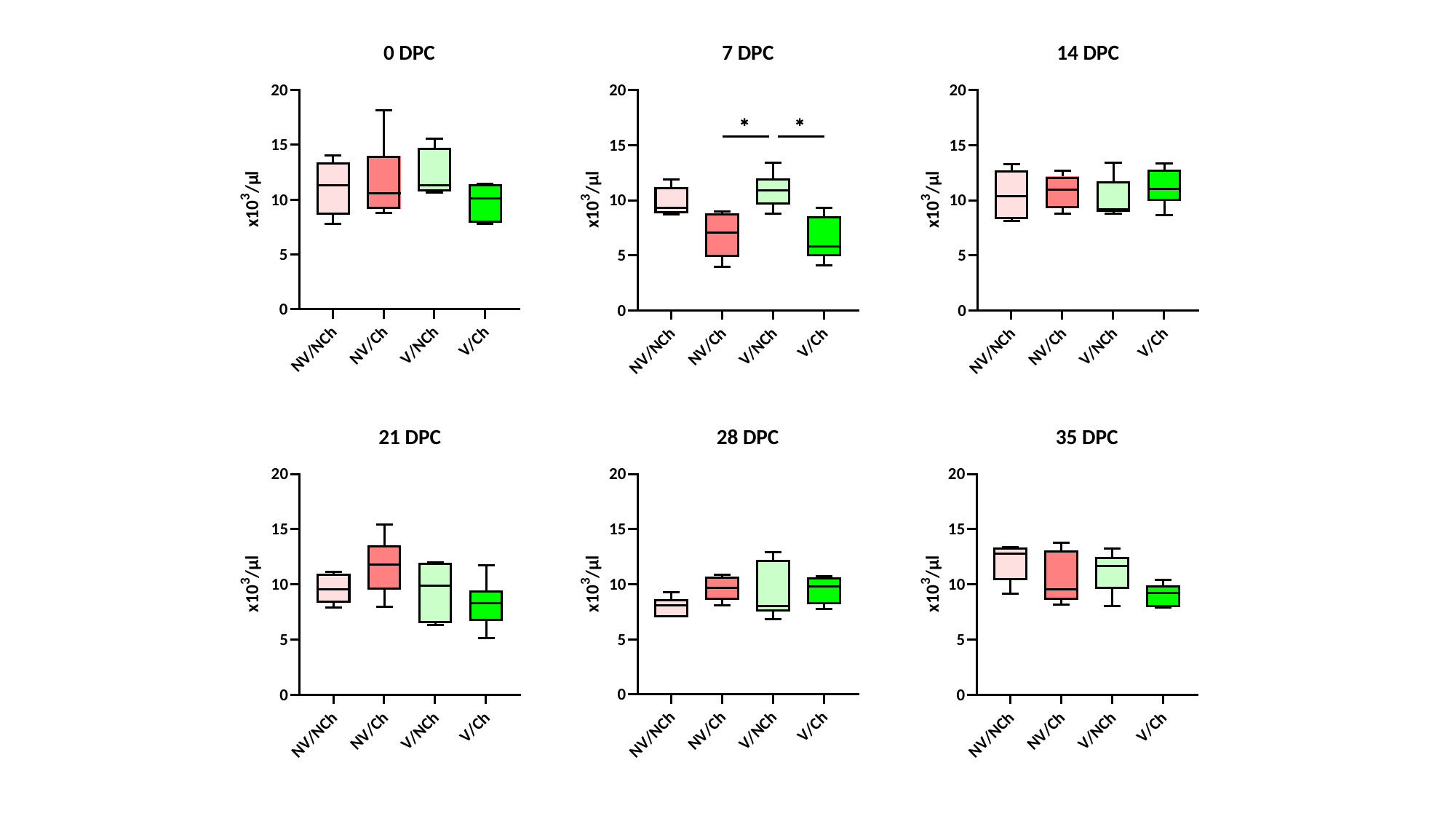

Supplement: Supplementary file 6 — Supplementary material 6. Evolution of lymphocyte counts in blood per group after challenge. The figure depicts weekly lymphocyte counts per group after challenge with statistical comparison between groups. Differences were only observed at 7 days post-challenge (DPC). V = vaccinated, NV= non-vaccinated; Ch = challenged, NCh= non challenged. *p<0.05 [file 40813_2025_423_MOESM6_ESM.pptx]

## Slide 1
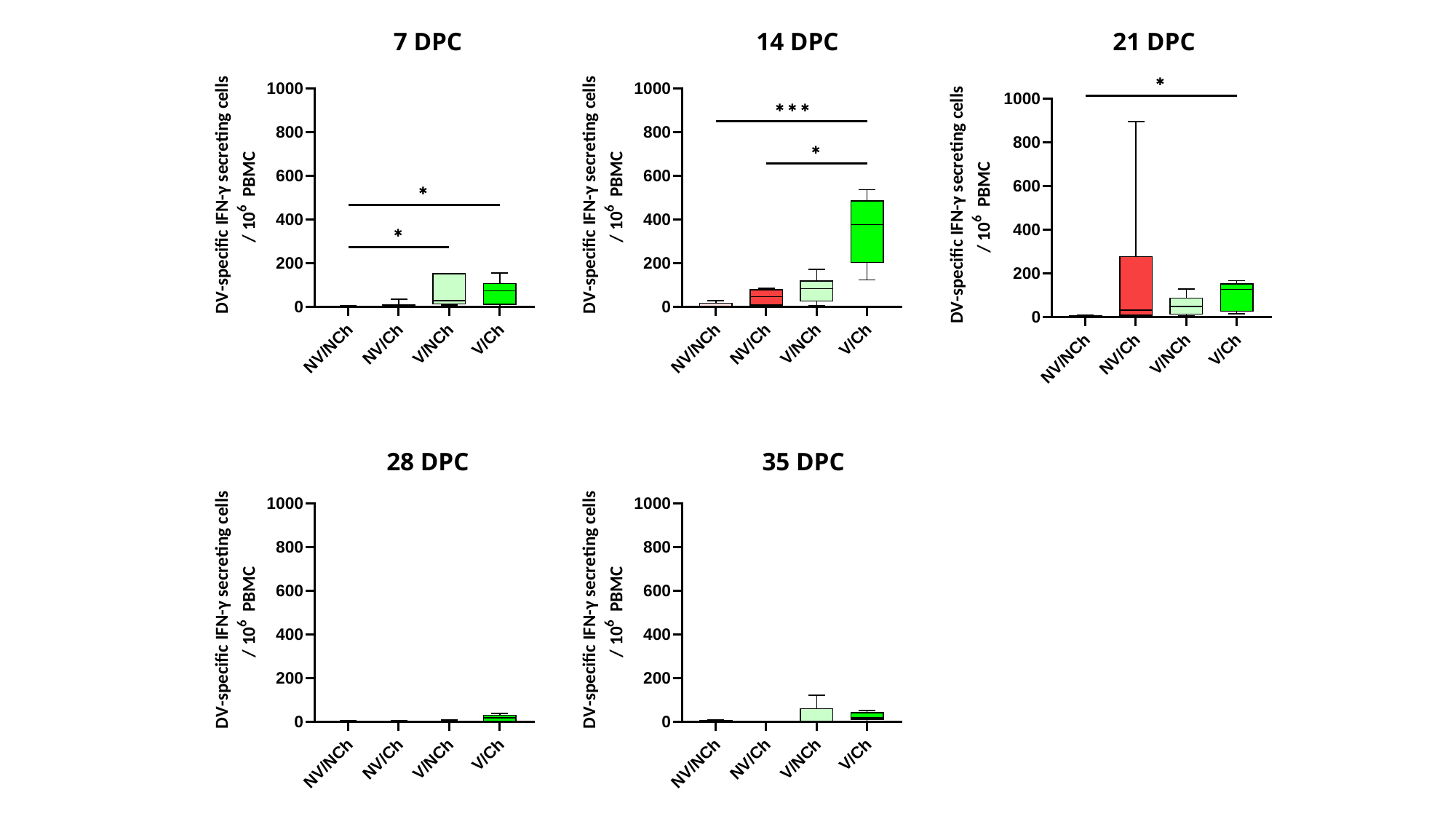

7 DPC
14 DPC
21 DPC
28 DPC
35 DPC

Supplement: Supplementary file 7 — Supplementary material 7. Evolution of the frequencies of IFN-γ secreting cells after challenge observed in ELISPOT using the vaccine strain as stimulus. V = vaccinated, NV= non-vaccinated; Ch = challenged, NCh= non challenged. *p<0.05; **p<0.01; ***p<0.001; ****p<0.0001 [file 40813_2025_423_MOESM7_ESM.pptx]
